# Pharmacological Management of Saddle Pulmonary Embolism in a High-Risk Patient With COVID-19

**DOI:** 10.7759/cureus.26211

**Published:** 2022-06-22

**Authors:** Megan D Biggs, Jonathan Bell, Christopher Park

**Affiliations:** 1 Internal Medicine, Rosalind Franklin University of Medicine and Science, North Chicago, USA

**Keywords:** interventional radiology guided embolization, critical care cardiology, submassive pulmonary embolism, covid coagulopathy, covid 19, saddle pulmonary embolism

## Abstract

A pulmonary embolism (PE) that is located in the main pulmonary artery is known as a saddle pulmonary embolism. Individuals at high risk who become unstable often require surgical intervention or more aggressive management with thrombolytic therapy. COVID-19 is a known risk factor for a hypercoagulable state and therefore increases the risk of PE and its associated complications. Individuals hospitalized with the COVID-19 virus and who have evidence of right ventricular dysfunction with PE are found to have a significantly higher risk of mortality. We present a case of an individual with several high-risk factors for PE as well as COVID-19 infection and evidence of cardiac strain, making the decision for treatment less clear. He was, however, treated successfully with heparin and enoxaparin alone. Furthermore, our case hadresolving symptoms of COVID-19, highlighting the importance of high clinical suspicion for PE in those diagnosed with COVID-19.

## Introduction

A pulmonary embolism (PE) is defined as an obstruction in the pulmonary artery due to a clot, tumor, air, or fat. A saddle pulmonary embolism is a clot within the main pulmonary artery that traverses the right and left pulmonary arteries [1]. Most individuals who are found to have a saddle pulmonary embolism, are hemodynamically stable, respond to treatment with unfractionated heparin and do not need thrombolytic therapy or surgical intervention [2]. However, various additional factors increase the risk of mortality in those with saddle PE, and the novel coronavirus SARS-CoV2 (COVID-19) has been found to be associated with significant PE risk [3]. We describe the case of a saddle PE in an individual with multiple PE risk factors and increased mortality risk who was successfully managed with heparin and enoxaparin.

## Case presentation

A 62-year-old male with a history of recurrent deep vein thrombosis (DVT) and pulmonary embolism since 2007, poor follow-up compliance, and on long-term rivaroxaban comes to the emergency department with right lower leg swelling for one week. He also reported new-onset dyspnea on exertion. He is unvaccinated against COVID-19 and three days prior to that he tested positive. Three weeks prior to presentation, he also developed fatigue, diarrhea, chills, and nausea with emesis that had resolved. Due to fatigue, he had been taking his anticoagulant inconsistently over the last three weeks. On arrival, he was found to have an oxygen saturation of 93% and a heart rate of 127. The revised Geneva score was 15 (indicating a high probability of PE) and the simplified pulmonary embolism severity index was indicative of high mortality risk due to increased heart rate. Electrocardiography revealed sinus tachycardia with right superior axis deviation. 

**Figure 1:**
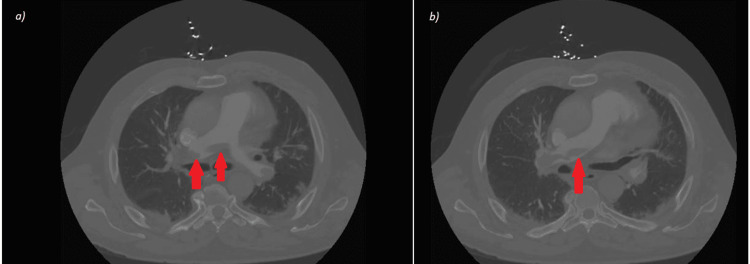
Cross-sectional views of CTA highlighting PE in red arrows. CTA: computed tomography angiography, PE: pulmonary embolism.

He was started on a heparin drip as well as remdesivir and dexamethasone. Significant laboratory values included a B-type natriuretic peptide (BNP) elevated to 1318, a negative troponin level, a D-dimer elevation of 6.73, and an international normalized ratio of 2.7. Prior coagulopathy workup in 2007 was negative for factor V Leiden, protein C and S, and antiphospholipid antibody syndrome. The physical exam was remarkable for erythema and edema extending from the right foot to the right buttock. Computed tomography angiography (CTA) showed extensive bilateral pulmonary embolus with additional saddle embolus, no clear evidence of right heart strain, as well as new patchy interstitial and airspace disease (Figure 1). A transthoracic echocardiogram was done, noting the right ventricular (RV) was not significantly dilated, the right ventricular systolic function was not significantly reduced, and the inferior vena cava was mildly dilated. The right ventricular to left ventricular (RV/LV) ratio was 0.9. Additionally, a venous duplex of the lower extremities showed extensive deep vein thrombosis (DVT) from the right popliteal vein proximally to the right common femoral vein as well as a lack of color flow in the right external iliac vein indicative of DVT. The patient was not a candidate for thrombolytic therapy as he was already taking therapeutic anticoagulation prior to admission. He was transferred to a tertiary center for thrombectomy of the lower extremity clot and evaluation for mechanical thrombectomy of the saddle pulmonary embolus. He remained hemodynamically stable and, therefore, his saddle PE continued to be managed with heparin infusion for five days and then enoxaparin for better inflammatory profile for four more days until discharge with symptomatic resolution.

## Discussion

Patients with submassive PE are at risk of becoming acutely unstable, and risk stratifying is important to determine treatment. Underlying comorbidities and lung conditions can decrease the ability to maintain hemodynamic stability. As many as 87% of individuals with saddle PE are hemodynamically stable. However, identifying those with higher mortality risk is imperative as saddle PE can be quickly fatal. Commonly used models to predict 30-day mortality are the Simplified Pulmonary Embolism Severity Indexes and the revised Geneva score, as noted in the above case. Likewise, elevated BNP, as seen in our case, can indicate right ventricular overload. Pulmonary embolism with contaminant DVT also increases the risk of mortality, as was also demonstrated in our patient [1]. One determinant of mortality and the need for possible surgical embolectomy when evaluating PE is right ventricular (RV) function. As many as 45% of individuals with saddle PE are hemodynamically stable, and RV strain has prognostic value in both stable and unstable patients [4]. Individuals demonstrating RV strain or instability can benefit from systemic thrombolytics or mechanical thrombectomy. Thrombolytic use reduces pulmonary vascular pressure and resistance, which restores RV function. Conversely, thrombolytics carry a risk of cranial hemorrhage. A surgical thrombectomy is an alternative option, especially for those who cannot undergo thrombolysis [5]. The FlowTriever Pulmonary Embolectomy Clinical Study evaluated patients with intermediate-risk PE who were treated with catheter embolectomy. Candidates were identified if they presented within 14 days of symptom onset and had RV strain indicated by a RV/LV ratio equal or greater than 0.9. The resulting reduction in RV/LV ratio was 25% with only a 0.9% risk of major bleeding [6]. While our patient qualified under these conditions, his RV/LV ratio was precisely 0.9, making the decision for embolectomy less clear.

Additionally, patients with COVID-19 have reported rates of symptomatic venous thromboembolism up to 25%. While the mechanism of COVID-19 clotting is unknown, multiple theories have been proposed that involve virus-infected endothelial cells causing possible damage with resulting vasculitis and platelet aggregation [3]. The COVID-19 virus is known to bind to angiotensin convertase enzyme 2. There is evidence this then leads to activation of extrinsic coagulation factors that result in increased thrombin and therefore enhanced platelet formation. Additional platelets create a greater risk of thromboembolic conditions such as PE [7]. There is also evidence that those hospitalized with COVID-19 and found to have RV dilation (defined by RV/LV ≥0.9 and/or RV diameter exceeding 4.1 cm) had a significantly higher risk of mortality [8].

## Conclusions

The patient, in this case, had several risk factors for PE, including prior history, anticoagulation noncompliance, current deep vein thrombosis, and COVID-19 infection. This case demonstrates successful anticoagulation management of saddle PE with intermediate-risk and elevated mortality based on several factors. Additionally, he remained stable while demonstrating right heart strain at the cusp of a significant RV dilation cutoff. This case further highlights the importance of high clinical suspicion for PE in those diagnosed with COVID-19, even in noncritically ill patients with improvement or resolution of COVID-19 symptoms.
